# Editorial: Community series in immunotherapy and small molecule inhibitors as combinational cancer therapeutics, volume II

**DOI:** 10.3389/fimmu.2025.1729774

**Published:** 2025-11-13

**Authors:** Mohd Wajid Ali Khan, Subhash K. Tripathi, Saravanan Rajendrasozhan

**Affiliations:** 1 Department of Chemistry, College of Sciences, University of Ha’il, Ha’il, Saudi Arabia; 2 Medical and Diagnostic Research Center, University of Ha’il, Ha’il, Saudi Arabia; 3 Center for Immunity and Immunotherapies, Seattle Children’s Research Institute, Seattle, WA, United States

**Keywords:** cancer, immunotherapy, immune check point inhibitors (ICIs), PD1/PD1-L, combinational therapeutics, digital treatment planners

The traditional methods of cancer treatments, such as surgery, chemotherapy, and radiotherapy, are not sufficient, and we need to focus on a new approach where these modalities are strategically combined with immunotherapy to unleash the full efficacy of the anti-tumor immune response. This transformation is shifting us from conventional treatments toward a future of a highly personalized and synergistic therapeutic era. The recent publications in the Research Topic “*Community Series in Immunotherapy and Small Molecule Inhibitors as Combinational Cancer Therapeutics: Volume II*” published in Frontiers in Immunology collectively provide a comprehensive overview of this evolution, elucidating the biological mechanisms, confirming clinical efficacy across major cancer types, and outlining the tools and novel targets that will define the future prospects in oncology care.

## 1 Radiotherapy—potential as systemic immune regulator

Development and innovation of novel therapies for advanced cancer are based on conventional treatments such as radiotherapies and chemotherapies, which lead to a diverse array of immune responses. Wang et al. showed evidence that radiotherapy (RT) can function as an *in situ* vaccine. It induces immunogenic cell death (ICD), releasing tumor antigens and damage-associated molecular patterns (DAMPs) that may initiate the activation of immune cells such as dendritic cells. Crucially, by causing DNA damage, RT activates the cGAS (Cyclic GMP-AMP synthase)-STING (Stimulator of interferon genes) pathway, leading to type I interferon production and inducing a robust T-cell response. This transformation of the tumor immune microenvironment (TIME) from an immunosuppressive state to an immunologically active state narrates the physiological phenomenon for the remarkable systemic tumor response, where localized irradiation results in the regression of metastatic lesions outside the radiation field. Whereas the same biological processes can induce immunosuppression, RT can upregulate checkpoint proteins like PD-L1, promote the expansion of regulatory T cells (Tregs) and myeloid-derived suppressor cells (MDSCs), and cause systemic lymphopenia, thereby counteracting its own immunostimulatory effects. This ensures that RT is not a passive partner but an active immune modulator. The clinical challenge, therefore, is to strategically harness its immunostimulatory potential while reducing its suppressive effect. This can effectively be achieved via a combination with immune checkpoint inhibitors (ICIs).

## 2 Efficacy of combination therapeutics in cancer treatments

An extensive study on advanced Non-Small Cell Lung Cancer (NSCLC) by Wang et al. showed that pembrolizumab with radiotherapy treatment enhanced the patient conditions and led to improved progression-free survival (PFS) and overall survival (OS), which is favorable compared to pembrolizumab alone, with notably enhanced distant tumor response rates.

This finding is strongly supported by the real-world study of Zheng et al., who developed a predictive nomogram for stage IV NSCLC. Data from 462 patients were collected in this clinical study exhibited the plan of the treatment was a key determinant of survival. Their findings provide evidence that chemotherapy in synergy with chemotherapy exhibited better outcomes than single therapeutic treatment. Further evidence of the success of the combinational therapeutics is exhibited by the meta-analysis studied by Sisodiya et al. for breast cancer. In their systematic review, they included 55 clinical trials that demonstrated that combination immunotherapies significantly improved both OS and PFS in all trial phases (I-IV) when compared with single therapy. The outcome from these clinical trials suggests that combinational therapies, which can include two or more treatment regimens such as RT, immune molecules, chemotherapy, etc., exhibited significant survival outcomes.

## 3 Testing novel immune molecules to enhance combinatorial therapeutics

The combinatorial therapeutics have had a significant effect on solid tumor treatments. A meta-analysis of phase III clinical trials conducted by Zhang et al. analysed the role of ICIs as first-line standard therapy for recurrent or advanced cervical cancer. The overall outcome of the study exhibited improvements in both progression-free survival (HR 0.67) and overall survival (HR 0.66) with ICI-based treatments compared to single therapeutic treatments. The positive outcome was observed in patients with higher expression of PD-L1 in tumors and those with histology of squamous carcinoma. The combination of ICIs with conventional therapies, however, was associated with a slight increase in adverse events (AVs) relative to standard therapy alone. These findings emphasize the importance of careful patient monitoring during combination therapy. This also sheds light on the need for a thorough assessment of toxicity risks before adopting such treatment strategies in clinical practice.

In a retrospective study, Wang et al. investigated the efficacy of combining the anti-angiogenic agent anlotinib with immune checkpoint inhibitors (ICIs) and platinum-based chemotherapy to improve outcomes in patients with non-small cell lung cancer (NSCLC). The triple combination therapy (AIC: anlotinib, ICI, and chemotherapy) achieved a median progression-free survival of 7.76 months, which was significantly longer (by 2.33 months) than that observed with the combination of ICIs and chemotherapy alone. These findings proved the significant role of adding anti-angiogenic agents to combination treatment regimens. Notably, even the two-drug combination of anlotinib and chemotherapy demonstrated superior progression-free survival compared with the ICI–chemotherapy regimen. These findings strongly suggest that for later-line NSCLC patients, the addition of an anti-angiogenic agent is critical to delaying disease progression. Furthermore, the authors reported that the overall risks and toxicities were tolerable and could be controlled. Although the study included a small sample size with single-center collection, the study showed the potential of triple therapy as an effective treatment option for NSCLC patients who have not responded to standard conventional treatments. Further randomized controlled trials are warranted to validate these findings and confirm the efficacy and safety of this therapeutic approach.

Li et al. carried out a retrospective study and compared the effectiveness of targeted immunotherapy vs targeted therapy alone in the third-line or beyond setting for microsatellite stable (MSS) metastatic colorectal cancer (mCRC) patients (n=71) to help identify the beneficial population of combined targeted-immunotherapy. Out of a total of 71 subjects, 31 received targeted therapies alone (TT group), and 40 received combinations of targeted therapy and immunotherapy (TI group). The outcome of the study was that combination therapy improved response rates (20% vs. 3.2%) and controlled disease (82.5% vs. 58.1%), with longer median progression-free survival (4.6 vs. 4.1 months). The most significant outcome of the combinational targeted immunotherapy was observed in patients with lung metastasis alone. These findings suggest that targeted immunotherapy combinations can enhance efficacy in selected MSS mCRC patients. Further studies with larger patient cohorts are still necessary to strengthen reliability and validity.

In another retrospective study involving 71 patients, Zhao et al. investigated whether baseline lymphocyte counts could help identify which hepatocellular carcinoma (HCC) patients would benefit from targeted combination immune therapy. The study showed that both progression-free survival (PFS) and overall survival (OS) improved (p = 0.058 and p = 0.077, respectively) in patients receiving combination therapy with tyrosine kinase inhibitors (TKIs) and PD-1 inhibitors. Notably, patients with a high peripheral blood lymphocyte count (PBLC) exhibited better OS and PFS as compared to the cancer patients with low absolute PBLC. These results highlight that PBLC could be a routine blood measure that can be used as a potential biomarker to identify HCC patients most likely to benefit from TKI and PD-1–based combination therapy. Implementing lymphocyte count as a stratification or decision-making tool could optimize precision therapy and minimize unnecessary toxicity and cost.

In a review published by Liu et al., the authors provided a wide overview of immunotherapeutic strategies for hepatocellular carcinoma (HCC), emphasizing various combination approaches. The authors discuss the clinical outcome of ICIs monotherapy and essential mechanisms by which ICIs activate immune cells and lead to the shift of immunosuppression in the tumor microenvironment towards immune activation. PD1 blockers such as nivolumab and pembrolizumab were found to be safe in the treatment of cancer patients. Both nivolumab and pembrolizumab exhibited lower efficacy for the HCC’s immunosuppressive tumor microenvironment, yielding objective response rates (ORRs) typically below 20%.

Consequently, therapeutic strategies have shifted toward combination regimens that synergistically enhance antitumor immunity and are now considered the standard of care. A clinical trial study published in 2018 showed that a combination of atezolizumab and bevacizumab introduced into unresectable HCC patients (n=104) resulted in a manageable safety profile with a PFS of 12.4 months, a median survival time of 17.1 months, an ORR of 36%, and a DCR of 71%. The landmark IMbrave150 trial established atezolizumab plus bevacizumab (“T+A”) as a first-line regimen, demonstrating a significant overall survival (OS) advantage over sorafenib. Additional trials, such as CARES-310 (camrelizumab plus apatinib) and HIMALAYA (durvalumab plus tremelimumab), also showed encouraging efficacy, with the latter achieving an ORR of 20.1%, median PFS of 3.8 months, and median OS of 16.4 months in unresectable HCC. Several studies have been conducted based on transcatheter arterial chemoembolization (TACE) in combination with ICIs for the treatment of unresectable advanced HCC patients. In addition, there is also the phase II study of TACE in combination with nivolumab for intermediate-stage HCC (IMMUTACE) and the phase III LEAP-012 (NCT04246177) study of TACE in combination with lenvatinib and pembrolizumab for intermediate-stage HCC, which also exhibited better results. However, this rapidly expanding combinational therapeutics involves significant challenges, including the need to identify optimal biomarkers for patient selection, manage unique immune-related adverse events, overcome primary and acquired resistance, and define the most effective sequences and combinations within an increasingly complex treatment landscape. The future of HCC therapy lies in deepening our understanding of the tumor-immune environment to guide these sophisticated, personalized combination approaches.

A meta-analysis study by Zhao et al. evaluates the safety and efficacy of combining concurrent chemoradiotherapy (CCRT) with ICIs in locally advanced cervical cancer (LACC). The combined data suggest that together, CCRT and ICIs may improve objective response rates (ORR) compared to CCRT alone, with an improved disease-free survival trend. Whilst these findings are promising, the evidence remains limited, and hence, long-term outcomes and overall safety require further investigation. This study emphasizes the potential of combining immunotherapy with standard LACC treatment to enhance therapeutic efficacy.

A systematic review and meta-analysis study evaluated the efficacy and safety of anlotinib in advanced digestive system neoplasms (DSNs). In total, 20 clinical trials, which included 1,613 patients, exhibited anlotinib combined with conventional cancer treatments significantly improved short-term outcomes. Overall patient survival time increased by 6 months. This study exhibited that the combinational therapy resulted in a higher incidence of adverse events, including hypertension, proteinuria, fatigue, and gastrointestinal disturbances. There were no treatment-related deaths. Subgroup analysis indicated a relatively less effect in advanced gastric cancer. These findings demonstrate anlotinib with other combinational interventions proved as promising therapeutics in DSN treatment (Zhou et al.). Furthermore, a more careful risk-benefit assessment is needed, and further studies must define long-term efficacy and optimal patient selection.

Nandi and Sharma showed the latest research relevant for the future directions of immunotherapy research and clinical trials: (a) destroying treatment-resistant cell populations through dendritic cell vaccines or CAR-T cells targeting Cancer Stem Cells (CSC) markers (e.g., CD44, EpCAM) is a promising strategy to prevent metastasis; (b) the presence, type, and functional state of tumor infiltrating lymphocytes (TILs) are important as prognostic and predictive biomarkers, and adoptive cell therapy using expanded TILs represents a highly personalized and potent treatment regime; (c) the gut and tumor microbiota are now recognized as potent regulators of immunotherapy response, and interventions like fecal microbiota transplantation (FMT) and specific probiotic/prebiotic regimens are being actively investigated to overcome primary and acquire resistance.

## 4 The imperative for personalization: the role of predictive modeling

With combinatorial therapeutics expanding to include immunotherapy with radiotherapy, chemotherapy, and other targeted agents, the clinical treatment decision-making process will become faster and more robust. The question is no longer merely whether to combine, but which agents to combine, for which patient, and in what sequence. The outcome of these strategies provides the transition from a one-size-fits-all approach to a deeply personalized treatment strategy. The work of Zheng et al. is a direct response to this need, developing a predictive nomogram for stage IV NSCLC that integrates patient-specific data to forecast individual survival probability. Such tools represent a favorable new era in clinical oncology.

Predictive models are essential for several reasons. First, they move clinical practice beyond population-level evidence, which is highly important for establishing efficacy. Heterogeneity of treatment effects suggests that individual patient responses to the treatment may vary. The therapy offers a modest survival benefit for one patient could be entirely ineffective for another. By including variables such as tumor genomics (e.g., PD-L1 status and mutational burden), clinical parameters (e.g., lactate dehydrogenase levels and sites of metastasis), host factors (e.g., baseline lymphocyte count as highlighted by Zhao et al. in HCC), and specific treatment conditions, these models can classify patients into subgroups most likely to derive benefit.

Second, these models are crucial for risk mitigation. As starkly illustrated by the case report of sintilimab-induced agranulocytosis by Qin et al., the potent activation of the immune system by ICIs carries the risk of severe and unpredictable toxicities. Predictive modeling is not solely about predicting efficacy; it is equally about identifying patients at high risk for immune-related adverse events (irAEs). A model that could flag a patient’s predisposition to hematological toxicity, for instance, would allow for enhanced monitoring and preemptive management, thereby improving safety.

The predicted future of these treatment tools relies on the development of a dynamic treatment plan designed by AI, using clinical data derived from the electronic health records of a diverse range of patients, multi-omics profiling, and even digital biomarkers. This continuous learning AI-designed treatment plan will enable the best use of available therapy and eventually create a “digital treatment planner” that can simulate the possible therapeutic outcome and side effects of various combinations of drug treatment for a particular patient. The data-driven treatment plan can ensure individual-specific cancer care and thereby maximize the therapeutic potential of a combination of drugs with minimal side effects.

## 5 Possible adverse effects of ICI

Qin et al. reported immune-related adverse events (irAEs) caused by a cancer immunotherapy drug, sintilimab (anti-PD-1 Ab). Sintilimab induced agranulocytosis in a patient with non-small cell lung cancer, which highlights the unpredictable side effects and limitations of ICI cancer therapy. Although ICI treatment is effective in cancer treatment, its mechanism of activating T-cells is primarily related to over-response of the immune system, leading to side effects like autoreactive immune responses, which can cause a life-threatening condition with severely low levels of white blood cells called neutrophils. Distinguishing the side effects of chemotherapy from irAEs is challenging and time-consuming. To treat the sintilimab-induced agranulocytosis, a high dose of corticosteroid was administered, which is not usually included in standard cancer care. The irAEs pose a significant clinical management challenge as they counterbalance the therapeutic benefits of ICIs.

## 6 Conclusion and future perspectives

The collective evidence confirms a major shift in oncology based on synergistic combinational therapies. We are moving decisively from the era of sequential, non-specific cytotoxic treatments to a synergistic era defined by rationally designed combination therapies that strategically harness and augment the host’s immune system. The combination of immunotherapy along with chemotherapy, radiotherapy, targeted agents, and/or localized treatments has become an effective clinical treatment strategy in treating various cancers, including NSCLC, breast cancer, HCC, and cervical cancer.

This new frontier, however, is accompanied by numerous challenges that need to be addressed for tailoring proper cancer treatment. As our therapeutic regimen expands, the principal challenge is the lack of robust, predictive biomarkers to guide selection among numerous combination options. The promising findings regarding baseline lymphocyte counts in HCC and PD-L1 status in cervical cancer are initial steps; the future demands the discovery and validation of multi-analyte signatures that can predict both efficacy and toxicity for specific drug combinations.

Optimizing treatment sequencing and timing has become crucial. The superior efficacy of neoadjuvant immunotherapy to some extent highlighted the importance of the treatment schedule. Choosing concurrent or sequential delivery in an optimal order of radiotherapy, chemotherapy, and immunotherapy is critical for maximizing synergistic potential and minimizing antagonistic effects.

To achieve the greater clinical benefit of innovative therapies, the management of irAEs is essential; this can be achieved by developing standardized, preventive management protocols and predictive models for irAEs.

Finally, the Research Topic of “easy access for everyone” must be focused on. The affordability of the multi-drug combination treatment is a significant barrier to widespread clinical use. Drug price control, by the combined efforts of researchers, clinicians, and policymakers, is essential to prevent disparity in cancer care. Looking forward, the future era of combinational therapeutics will exploit artificial intelligence and multi-omics data to create dynamic and individualized “digital treatment planners” based on the clinical effectiveness of the drugs with respect to the patient-specific factors.

In conclusion, combinational treatment designed with a multi-target approach on the tumor-immune ecosystem will be the future of standard cancer treatment. This promising therapeutic approach has the potential to significantly improve the quality of life and survivability of cancer patients, effectively transforming cancer into a more manageable disease.

